# Verbal autopsy interpretation: a comparative analysis of the InterVA model versus physician review in determining causes of death in the Nairobi DSS

**DOI:** 10.1186/1478-7954-8-21

**Published:** 2010-06-29

**Authors:** Samuel O Oti, Catherine Kyobutungi

**Affiliations:** 1 African Population and Health Research Center, P.O. Box 10787 GPO-00100, Nairobi, Kenya

## Abstract

**Background:**

Developing countries generally lack complete vital registration systems that can produce cause of death information for health planning in their populations. As an alternative, verbal autopsy (VA) - the process of interviewing family members or caregivers on the circumstances leading to death - is often used by Demographic Surveillance Systems to generate cause of death data. Physician review (PR) is the most common method of interpreting VA, but this method is a time- and resource-intensive process and is liable to produce inconsistent results. The aim of this paper is to explore how a computer-based probabilistic model, InterVA, performs in comparison with PR in interpreting VA data in the Nairobi Urban Health and Demographic Surveillance System (NUHDSS).

**Methods:**

Between August 2002 and December 2008, a total of 1,823 VA interviews were reviewed by physicians in the NUHDSS. Data on these interviews were entered into the InterVA model for interpretation. Cause-specific mortality fractions were then derived from the cause of death data generated by the physicians and by the model. We then estimated the level of agreement between both methods using Kappa statistics.

**Results:**

The level of agreement between individual causes of death assigned by both methods was only 35% (κ = 0.27, 95% CI: 0.25 - 0.30). However, the patterns of mortality as determined by both methods showed a high burden of infectious diseases, including HIV/AIDS, tuberculosis, and pneumonia, in the study population. These mortality patterns are consistent with existing knowledge on the burden of disease in underdeveloped communities in Africa.

**Conclusions:**

The InterVA model showed promising results as a community-level tool for generating cause of death data from VAs. We recommend further refinement to the model, its adaptation to suit local contexts, and its continued validation with more extensive data from different settings.

## Background

Developing countries generally lack consistent, timely, and reliable information on the levels and cause of death patterns in their populations [1]. Information about causes of death is needed by health managers and policymakers at every level of governance -from local to national - to plan for, prioritize, and address the health needs of their people [2]. In developed countries, this information is usually made available through well-established vital registration systems [3]. For most of the developing world, where already scarce resources need to be carefully and optimally allocated, vital registration systems are weak, and information on causes of death is often incomplete or nonexistent [1,2].

Alternative sources of cause of death (COD) information come from Demographic Surveillance Systems (DSS), which in the last 10 years have been receiving increased attention for their ability to provide invaluable field data on mortality patterns in developing countries [4,5]. DSS monitor and track demographic and health indicators in a population within a defined geographical area [6]. Typically, DSS record vital events of births, deaths, and migrations within the population under surveillance at regular intervals ranging from quarterly to annually. Although DSS collect information from defined populations, the vital data that they generate can be linked to similar data from other DSS or sample registration systems within the same country or region to produce more representative data [7]. For instance, INDEPTH - the International Network of field sites with continuous Demographic Evaluation of Populations and Their Health - has produced a monograph series that presents comparative age-specific mortality patterns for INDEPTH field sites across Africa and Asia [8]. Such information has been used successfully for health planning in resource-constrained settings. For instance, in Tanzania, COD data were utilized for district and national health planning and resulted in reductions in child mortality [9]. Data from Health and Demographic Surveillance Systems have also been used to produce life tables for developing countries [8] and to estimate regional and global disease burdens [10-12]. Hence, DSS play an important role in producing critical information that can be used for planning and management in developing countries that lack routine vital registration systems.

DSS commonly use a methodology known as verbal autopsy (VA) to generate COD data. The process entails interviewing the primary caregivers of recently deceased persons to gather information on the circumstances surrounding the death [13]. It is based on the premise that the primary caregiver - usually a family member - can recall, volunteer, and recognize symptoms experienced by the deceased that can be interpreted later to derive a probable cause of death [14]. The purpose of VA is to describe the cause of death structure at the population level rather than to diagnose the cause of death at the individual level.

A VA data collection instrument is used to conduct the VA interview. This instrument is usually a questionnaire with an open-ended/narrative section for recording a verbatim account of the circumstances surrounding death and a closed section with filter questions of symptoms and signs of disease and/or injury [15]. There are numerous and diverse VA questionnaires used by various DSS and sample registration systems, but recently, there have been attempts to harmonize these tools internationally [13,14,16]. Specifically, recent efforts by the World Health Organization (WHO), INDEPTH, and other partners to standardize the VA data collection process have resulted in the elaboration of key characteristics of VA data collection instruments [13,15].

Apart from the various tools used for collecting VA data, there are also different methods for interpreting these data to derive probable causes of death. These include physician review, algorithms, and use of neural networks [14]. Physician review (PR) is the most commonly used method and typically involves the independent review of VA data by one or more local physicians. These physicians assess each completed VA questionnaire and, using the International Classification of Diseases Version 10 (ICD-10) list or an abridged version, assign the single most probable cause of death to each case [17-22]. There have been various attempts at validating PR, [19,21] but there are several concerns that arise from using this methodology to interpret VA data. First, physicians may differ systematically in their methods of interpreting VA data based on their training, experience, and/or perceptions of local epidemiology. Hence, there may be inter- and intra-coder variability between physicians that may lead to inconsistencies in COD data and also hinder reliable temporal and spatial comparisons of mortality [23,24]. Second, the PR process often demands a considerable amount of physician time and can incur considerable costs for remunerating these physicians.

Consequently, various alternative methods to physician review of VA data have been introduced. These include the use of expert/data-driven algorithms, neural networks, and a computer-based probabilistic model known as InterVA (Interpreting Verbal Autopsy). Algorithms and neural networks are said to have the advantage of being quicker, more transparent, and more consistent in comparison to PR [20,25,26]. However, both methods have been explored inconclusively in terms of their validity, and thus their use is still not widespread [20,26]. The use of the InterVA model to interpret VA data is a relatively new methodology that recently has been successfully explored in a number of settings. This computer program is based on Bayes' probability theorem and is said to have the advantage of achieving maximum consistency in interpreting VA data [27-29]. It also requires minimal time and labor resources, especially in comparison to the PR method. Moreover, it is freely available in the public domain, making it ideal for resource-constrained settings [30].

The aim of this paper is to explore how the InterVA model performs in comparison to PR in interpreting VA data collected by the Nairobi Urban Health and Demographic Surveillance Site (NUHDSS). Since its inception in 2002, the NUHDSS has relied on PR to interpret its verbal autopsy data, and this paper therefore seeks to determine the suitability of the InterVA model as an alternative to physician interpretation of VA data.

## Methods

### Study Area and Population

Since 2002, the African Population & Health Research Center (APHRC) has been operating the NUHDSS. The NUHDSS covers the two urban informal settlements (slums) of Korogocho and Viwandani, both located about 5 to 10 kilometers from Nairobi, the capital city of Kenya. Viwandani and Korogocho each occupy an area of 0.45 and 0.52 km^2^, respectively, and are inhabited by about 60,000 people from more than 15 ethnic groups. The population in Viwandani is mainly comprised of labor migrants working in the neighboring industrial area, while that of Korogocho is mainly comprised of long-term settlers engaged in the informal sector. These slum settlements, like most others in Nairobi, are characterized by relatively high crime rates, drug and alcohol abuse, risky sexual behaviors, high unemployment rates, poor access to health facilities, low school participation, and extreme poverty compared to other urban residents as well as their rural counterparts [31]. Data on individual and household core demographic events (birth, death, in-migration, and out-migration) in the two slums are collected at four-month intervals - also known as data collection rounds. In addition to the routine data collection rounds, the DSS also integrates the VA process for COD ascertainment.

### Verbal Autopsy

Deaths are usually identified during the DSS data collection rounds by trained field interviewers who complete a one-page death registration form (DRF) and then inform their field supervisors about the deaths. Supervisors are experienced field interviewers who have a minimum qualification of a bachelor's degree. They conduct the VA interviews using a VA questionnaire developed in conjunction with other INDEPTH sites. This questionnaire has two formats: one for deaths of children less than 5 years of age and the other for deaths of persons 5 years and older. The latter has an additional section on maternal deaths. The questionnaire covers the background characteristics of the deceased and the respondent as well as structured filter questions on specific signs and symptoms experienced by the deceased up to the point of death. There is also an open section that allows for recording of a narrative account of the events leading to the death. On average, it takes about 30-45 minutes to administer the VA questionnaire.

Before a VA interview is conducted, the field supervisor visits the household in his/her zone where a death has occurred as soon as he/she learns of the event and consoles the bereaved family. He then assesses the situation and decides whether the timing is appropriate to conduct the interview. If it is not appropriate, he/she makes an appointment with the family to return at an agreed later date - usually three to four weeks later. However, VA interviews may be conducted as long as six months after death due to operational reasons. At the first visit, the field supervisor identifies a "credible respondent" - usually a spouse or relative - who will participate in the interview. If the deceased is a child, the preferred credible respondent is usually a parent. Several revisits may be made to the household until a credible respondent is identified. After five such visits or if it is established that the remaining household members are no longer residents of the area, a credible neighbor is interviewed if he/she is willing. Otherwise, the verbal autopsy is coded as missing, and no cause of death is assigned to such cases.

### Interpretation of VA questionnaires

All completed VA questionnaires are collated and sent to three local physicians for interpretation. At least one of the three physicians is a full-time researcher employed with APHRC, while the other two are consultants or medical officers in public or private practice who review VA data on a contractual basis. Each physician independently reviews all the VA questionnaires and assigns a single COD based on ICD-10. The complete ICD-10 list has 12,420 unique codes for diseases, signs, symptoms, abnormal findings, complaints, social circumstances, and external causes of injury [32]. Hence, for practical purposes, the physicians use an abridged version of the ICD-10 list modified in such a way that uncommon causes of death in the study area are collapsed into broader categories. This modified list has 60 codes for possible COD (see additional file 1). If two of the assigned COD for each VA questionnaire are identical, this is taken as the final COD for the deceased. However, if all three of the assigned COD are different, the physicians hold a consensus meeting and review the case. In cases where consensus is not reached at these meetings, the COD is classified as "indeterminate."

### The InterVA model

The InterVA model is a probabilistic model based on Bayes' theorem that seeks to define the probability of a cause (C) given the presence of a particular indicator (I), represented as P(C|I). This probability can be stated as:

where P(!C) is the probability of not (C). Therefore, for a set of VA symptom-level data or indicators (I1...In) and for each possible cause of death resulting from these indicators (C1...Cm), there is an associated indicator Ij and cause Ck, whose probability of occurrence at population level can be determined. For each case, therefore, the probability of Ck is initially the value found among all deaths in total, which gives the cause-specific mortality fraction. For each case and each applicable indicator, however, the above theorem can modify the probability of Ck. Thus, the VA model adjusts the probability of each likely cause according to a matrix of P((I1...In)|(C1...Cm)) and then produces a summary listing of as many as three possible causes and their corresponding likelihood values [28]. Full details of the InterVA model and how it was developed based on the above theorem have been described in previous studies [27-29]. The model is run using computer software -Visual FoxPro - that provides a user interface into which a set of 100 indicators must be entered for each VA case in order for the model to generate a COD. These indicators are basically specific information comprising reported symptoms, signs, and medical history that need to be extracted from completed VA questionnaires. Some examples of the required indicators include: "Any difficulty in breathing?" "Any weight loss?" "Any coughing with blood?" Thus, when the model is run on these indicators, it automatically generates a listing of any of 30 probable COD for each verbal autopsy case (see additional file 1 for COD listing). A maximum of three probable causes of death and their corresponding likelihoods (in percentages) are presented in the list.

Additionally, the InterVA model has a built-in facility to adjust for the prevalence of malaria and HIV/AIDS in any setting such that before running the model, the prevalence of HIV/AIDS and malaria in the study population can be set as high or low. This was introduced during a process of refining the original model to address underlying conceptual issues of VA data collection and interpretation. The Delphi technique using a panel of experts was utilized to develop consensus on key conceptual issues of cause of death classification and VA usage, including adjustment for large variations in the prevalence of malaria and HIV/AIDS at the population level between regions. This adjustment significantly improved the performance of the model and increased the model's potential to be applied in different settings [27,28]. Details of the methods and how the built-in facility was developed are beyond the scope of this paper. For our study, we set the prevalence of malaria to be low and that of HIV/AIDS to be high. Previous research in our study population has demonstrated that the prevalence of malaria within this population is less than 0.5% [33], while HIV/AIDS prevalence is as high as 12.4% [APHRC 2008, unpublished data].

### Data analysis

Between August 2002 to December 2008, 1,823 VA questionnaires were reviewed by physicians who assigned a COD to each case. The required indicators from each of these questionnaires were extracted and entered into the InterVA model to automatically generate COD.

There are a total of 60 possible causes of death assigned by physicians, while the InterVA model only assigns 27 causes (see additional file 1). Therefore, to allow for meaningful comparison between physicians and the model, we re-categorized all causes in both methods into 14 main groups of causes for two reasons. First, we took this step to have comparable cause of death categories between both methods being analyzed. Where possible, we retained the categories common to both methods. For instance, malaria and meningitis, which are common to both physician review and InterVA, were retained as stand-alone causes. In cases where there were no direct correlates, we had to collapse and/or re-categorize the causes of death into cause groups to match each other in a broad sense. For instance, the InterVA model has only one broad category of maternity-related deaths representing all types of pregnancy-related deaths. However, the physicians coded causes such as eclampsia and ante-partum and post-partum hemorrhage. Such causes were therefore recoded into one broad category of maternity-related deaths so we could compare with the corresponding InterVA category. Frequently occurring conditions, such as pulmonary tuberculosis, HIV/AIDS, and pneumonia, were left as stand-alone causes. Second, it was more important to us that the model and the physicians arrived at broad agreement in identifying cause of death groups with the greatest public health importance at population level, rather than individual-level causes. Hence, causes such as kidney disease and cancers were recoded as chronic diseases, while causes such as rabies, tetanus, and typhoid were grouped into other acute/infectious diseases.

We then determined the cause-specific mortality fractions (CSMF) of using the InterVA model and physician review. We conducted our analysis for the general population and by two main age groups: children aged less than 5 years and for adults aged 18 years and older. While it is possible to conduct the analysis across various age categories, we decided to focus on the under-5 and adult deaths due to high levels of mortality in these age groups from preventable conditions such as diarrheal disease and HIV/AIDS, respectively [34]. Such preventable conditions are of great public health significance, especially in developing countries. Thereafter, we estimated the level of agreement between InterVA and physician-assigned COD using Kappa statistics. All analyses were carried out using STATA version 10 statistical software. In all our analyses, we only considered the most probable COD assigned by the model rather than all three possible causes. This is because the COD assigned by the physicians included only a single cause of death.

## Results

A total of 1,823 VA interviews were successfully completed and reviewed by physicians for the period August 2002 to December 2008. Children aged less than 5 years accounted for 572 cases (31.4%), and adults aged 18 years or older accounted for 1,166 cases (64%). Of the under-5 deaths, 384 (67%) occurred before the first year of life. Overall, males accounted for 1,020 (56%) deaths, and Korogocho had the majority (66%) of deaths of the two study areas.

For the 1,823 deaths, physicians successfully assigned a single cause in 1,443 (79%) cases on the first attempt. After holding consensus meetings, they successfully assigned single causes of death to an additional 252 (14%) cases. Therefore, in total, physicians assigned a single cause of death in 1,695 cases (93%). No consensus was reached in 128 cases (7%), which were coded as "indeterminate" by the physicians. The InterVA model assigned a single or primary cause of death in 1,290 cases (70.8%), two causes of death in 152 cases (8.3%), and three causes in six cases (0.3%). In 375 cases (20.6%), the InterVA model assigned the cause of death as "indeterminate."

A direct comparison of the causes of death assigned by the physicians to the primary causes of death assigned by the InterVA model (Table 1) shows that overall, there was direct agreement in 630 cases or 35% (κ = 0.27, 95% CI: 0.25 - 0.30). If all "indeterminate" cases were dropped, the level of agreement increased to 47% (κ = 0.40, 95% CI: (0.36 - 0.42). The level of agreement was 32% for deaths in children aged less than 5 years and 36% for adults 18 years and older.

**Table 1:** Summary of case-by-case agreement between PR and InterVA

	Cases (deaths) in agreement	Kappa (95%CI)
Children aged less than 5 years	181 (31.64%)	0.224 (0.183 - 0.253)
Adults (18 years and older)	204 (35.59%)	0.254 (0.222 - 0.282)
Overall	630 (34.56%)	0.271 (0.247 - 0.293)

CI, Confidence Interval.

We did further analysis to compare the level of agreement between the physicians and the InterVA model for the 152 cases that had two likely causes of death assigned by the model. We did not analyze cases with three likely causes because they were too few (only six cases). Our results showed that among the 152 cases, the level of agreement was 31% (κ = 0.23, 95% CI 0.19 - 0.25) between the physicians and the primary cause assigned by the model. However, the level of agreement dropped to 26% (κ = 0.17, 95% CI 0.11 - 0.25) if we compare the physicians' causes to the second most likely causes of death as assigned by the model.

**Figure 1:**
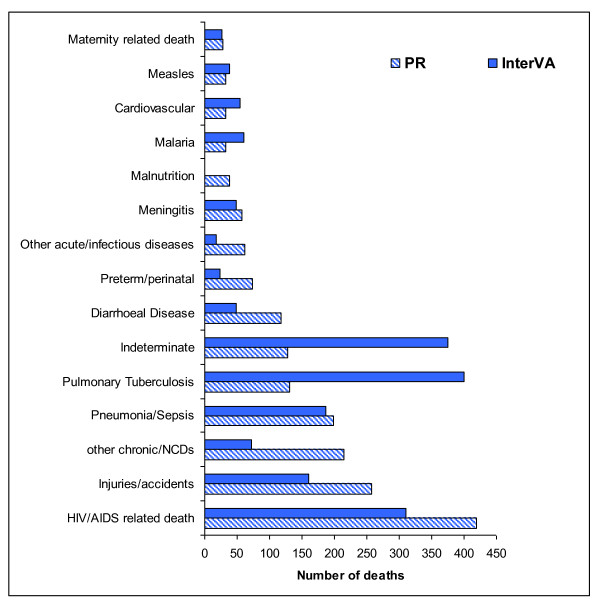
**Representation of the major cause of death categories derived from physician review and InterVA model interpretation of 1,823 deaths in the NUHDSS, 2002 -2008**.

Figure 1 shows the major COD categories and the numbers of deaths in each category as assigned by physicians and the InterVA model. Overall, the majority of deaths were attributable to infectious diseases, including pulmonary tuberculosis, pneumonia, and HIV/AIDS. Specifically, 1,051 cases (58%) were attributed to infectious causes by physicians and 1,109 cases (61%) by the InterVA model. However, 249 cases (14%) were attributed to noncommunicable diseases by physicians, while only 126 cases (7%) were attributed as such by the InterVA model. Physicians assigned 257 cases (14%) to injuries, while the model assigned this cause to 127 cases (8.8%). It is noteworthy that the InterVA model attributed significantly more causes of death to pulmonary tuberculosis (401 cases) compared to physicians (131 cases). However, physicians attributed more causes of death to HIV/AIDS (419 cases) compared to InterVA (310 cases).

**Figure 2:**
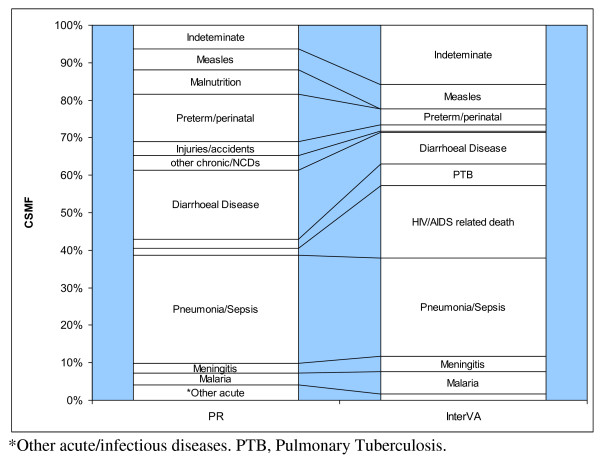
**Cause-specific mortality fractions for 572 deaths of children aged less than 5 years in the NUHDSS from 2002-2008, derived from verbal autopsies interpreted by physicians and by the InterVA model**.

For children aged less than 5 years, there were 572 deaths assigned causes by both physicians and the model. Figure 2 shows the CSMF as assigned by physicians and the model. Of all deaths in this age group, the majority (382 cases or 67%) was due to infectious diseases as assigned by physicians. An even greater number of cases (446 or 78%) were attributed to infectious diseases by the model. A notable difference between the two methods is the frequencies with which deaths in this age group were attributed to HIV/AIDS. The InterVA model attributed 110 out of 572 (19.2%) deaths to HIV/AIDS, whereas the physicians only attributed 10 (1.7%) deaths to HIV/AIDS. On the other hand, physicians attributed 105 (18.4%) deaths to diarrheal diseases, while the model attributed only 48 (8.4%) deaths. Also, the model assigned twice as many deaths to pulmonary tuberculosis as the physicians. Other infectious diseases such as pneumonia, malaria, and measles only showed slight differences in proportions assigned by the two methods. Injuries and noncommunicable diseases accounted for less than 8% of deaths as assigned by both methods.

There were notable differences in the proportions of deaths assigned to preterm/perinatal conditions and malnutrition. There were three times more deaths (72 cases) attributed to preterm/perinatal causes by physicians in comparison to the model (24 cases). Also, there were 37 cases attributed to malnutrition by physicians, while the model did not assign any cause of death to malnutrition. Among deaths to children less than 5 years old, the model arrived at an "indeterminate" cause in 90 cases (15.7%), while physicians did the same in 37 cases (6.3%).

**Figure 3:**
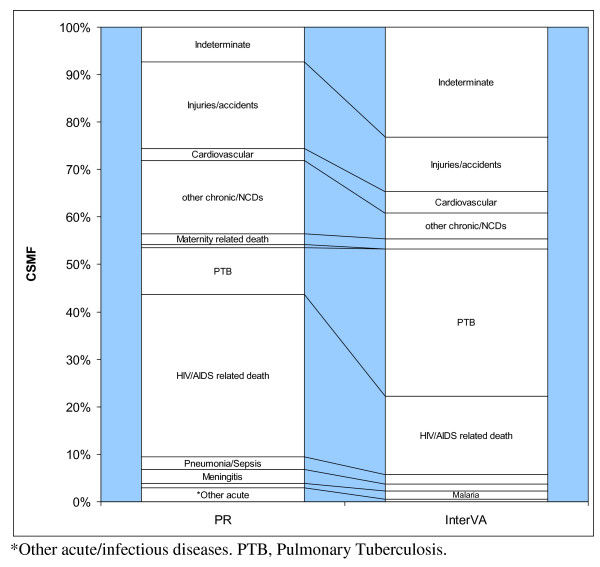
**Cause specific mortality fractions for 1,166 adult deaths in the NUHDSS from 2002-2008, derived from verbal autopsies interpreted by physicians and by the InterVA model**.

For the 1,166 deaths in persons aged 18 years and older, we found that infectious causes accounted for just more than half of all deaths as assigned by physicians (54%) and the model (53%). The main difference again lies in the proportions of deaths attributed to tuberculosis and HIV/AIDS (see Figure 3). Physicians assigned 401 cases (34%) to HIV/AIDS and 117 cases (9.9%) to pulmonary tuberculosis (PTB). However, the model assigned 194 cases (16%) to HIV/AIDS and 365 cases (31%) to PTB. We found that physicians attributed twice as many deaths to noncommunicable diseases as the model - that is, 18% and 9% respectively. A difference of similar magnitude was also found in deaths attributed to injuries - that is, 19% attributed by physicians and 11.7% by the model. Finally, we found that physicians arrived at an "indeterminate" cause in 86 cases (7%), whereas the model did so in 275 cases (23%).

## Discussion

The level of agreement between physician review and the InterVA model was less than 40% of the 1,823 deaths interpreted by both methods. Other similar studies have shown higher levels of agreement, ranging from 50% to 83%, albeit with much smaller sample sizes [27-29]. However, it is encouraging that the overall picture of CSMF for the major causes of death in our study population was somewhat similar as determined by both methods. This is despite the fact that the InterVA model was applied independently and at a single time point to VA data that had been previously reviewed by different physicians at various time points over a period of almost eight years. Even more encouraging is the finding that the patterns of mortality data generated by both methods were consistent with those found in previous studies on the burden of mortality in slum populations and other disadvantaged populations in less developed countries [34,35].

However, there are several differences and contradictions in the assignment of causes of death by either method that need to be considered. First, we found that the InterVA model more frequently identified pulmonary tuberculosis as a cause of death than was done by physicians. On the other hand, physicians more frequently identified HIV/AIDS as a cause of death than the model. This is not entirely surprising as there is a great deal of overlap between both disease conditions in terms of clinical symptoms and signs [36]. Furthermore, the re-emergence of pulmonary tuberculosis in several countries of the world is spurred by the HIV/AIDS pandemic [37]. This underlies the high level of interconnectedness between both diseases. Moreover, from a public health perspective, control and prevention of either disease cannot be considered without regard to the other [38]. Hence, what is critical is that the collective burden of both diseases in any population is clear, and the InterVA model achieved this as successfully as the physicians did.

As regards noncommunicable diseases, we found that physicians identified this category as a cause of death almost twice as often as the model. Further inspection of the data revealed that one-quarter of the physician-assigned noncommunicable disease deaths were actually identified as PTB or HIV/AIDS by the model (see additional file 2). A slightly larger proportion was identified by the model to be "indeterminate." A similar magnitude of differences was also observed in the identification of deaths due to injuries. Again, physicians assigned more than twice the number of injuries as the model. However, unlike the noncommunicable disease cases, the model settled for a diagnosis of "indeterminate" in virtually all cases that differed from the physicians' diagnoses of injury. The reasons for this difference in coding noncommunicable diseases are not immediately clear, and it is difficult to say whether or not the physicians' diagnoses were more appropriate than the model's. However, for the injuries, it is clear that the model did not have enough information to arrive at a diagnosis and simply settled for indeterminate. This is largely due to the design of the VA questionnaires, which are structured in such a way that the richest source of information on the circumstances surrounding injuries is the narrative section of the questionnaire. The structured/close-ended part of the questionnaire is more suited for symptoms and signs of disease rather than circumstances leading to injury. Hence, most of the completed VA questionnaires for cases of injury have very sparse details in the structured part. Physicians may therefore have benefited from reading the open narrative section of the VA questionnaire as this would certainly have contained more information on injuries than what was captured in the close-ended part of the questionnaire.

In children younger than 5 years of age, we find that the model identified HIV/AIDS as a cause of death at a much higher frequency than physicians, who mostly diagnosed such cases as either diarrheal diseases or malnutrition. A similar finding has also been demonstrated in previous studies. The explanation given in these studies is that considering that malnutrition and diarrheal disease are major causes of under-5 mortality in poor and undeveloped settings [39], it is likely that the model overestimated HIV/AIDS prevalence in children, while physicians may have underestimated it. We suspect that a similar possibility may have occurred in our study. However, we cannot fully explain why the model did not assign any cause of death to malnutrition. We observed that the majority of the cases of malnutrition diagnosed by physicians were identified as HIV/AIDS by the model. Specifically, of the 37 cases of malnutrition diagnosed by physicians, the model assigned 18 cases to HIV/AIDS, five to tuberculosis, four to pneumonia, four to malaria, and six to other conditions. This is not surprising considering that symptoms such as chronic diarrhea and weight loss are common to some of these conditions as well as to malnutrition. Furthermore, most of these conditions are very likely to either co-exist with or complicate malnutrition. Also, our experience in VA interpretation suggests that there is weak reporting of certain key features of malnutrition such as abnormal hair changes, abdominal swelling, and edema that may have impaired the model's ability to arrive at this diagnosis. It may therefore be easier for physicians to spot malnutrition as an underlying cause of death, especially through the additional benefit of the narrative section of the VA data not considered by the model.

Other differences in the interpretation of VA data by physicians and by the model include assignment of "preterm/perinatal" deaths in children by either method. We observed that about one-third of the cases identified by physicians as "preterm/perinatal" deaths were assigned as "pneumonia/sepsis" by the model. This finding was similar to what was observed in another study in a rural community in Ethiopia [29]. Just as in that study, we believe that the reasons for our observed differences in interpretation arose from differences in definitions of this category of deaths. Specifically, it should be emphasized that, for ease of analysis, the term "preterm/perinatal" was used broadly to include all causes of death in the early neonatal period, including conditions such as birth asphyxia, birth injuries, congenital deformities, neonatal sepsis, and neonatal jaundice, among others. Furthermore, it should be noted that the InterVA model's age grouping defines "< 4 weeks old" as its lowest age group. This means that unlike physicians, the model is unable to distinguish deaths in the perinatal period (first seven days of life) from other neonatal deaths.

The low level of agreement between physician review and the InterVA model in this study highlights several limitations of either method. As far as InterVA is concerned, it is important to reiterate that the model has a pre-defined set of indicators that it depends upon to arrive at causes of deaths. These indicators need to be extracted from the VA data and fed into the model. Some of these indicators were not available in the VA data as there were no specific questions in the VA questionnaires that collected information on these indicators. For instance, the VA questionnaires do not contain questions on the history of the deceased's alcohol or tobacco use, raised or lowered fontanelles, symptoms or signs of herpes, excessive thirst, excessive hunger, history of liver disease, or history of sickle cell anemia or cancer. Also, there are several questions in the VA questionnaires that were not built into the InterVA indicator set. For instance, the VA questionnaires have useful questions pertaining to drug/treatment history and health service utilization. It is therefore unclear how the absence of the above information may have affected the performance of the InterVA model.

With regard to physicians, we must consider that, as previously mentioned, they have the added advantage of being able to utilize the narrative section of the VA questionnaires. The narrative section often contains redundant details that physicians have to sift through to get the most relevant facts pertaining to the death under review. The InterVA model can only utilize the narrative section if key words from the text are manually extracted and fitted into the model. For a large number of VA questionnaires, this would be a time-consuming process that would preclude the argument that the model is a time-saving alternative to PR. Also, the physicians are able to use their clinical skills and experiences to make a final judgment between disease entities that may have strikingly similar symptoms and signs. However, physicians may also be influenced by their own experience-driven biases that have traditionally raised issues of inter-observer reliability and the hindrance that this poses to temporal and regional comparisons of COD. Furthermore, it is clear that PR of VA data is not a "gold standard," and this has raised concerns about validating other interpretation methods against it. It is precisely for this reason that we have avoided using measures such as sensitivity, specificity, and positive predictive value in our comparative analysis of the InterVA model against PR data. Additionally, while it would have been ideal to validate the model with respect to hospital COD as "gold standard," as has been done in other studies [17,19] we could not do so in our study. This was because the majority of deaths in the slums occur outside hospital settings (about 80% in the NUHDSS). Hence, the deaths that occur in a hospital may differ selectively from those outside it, and this will inherently bias the validation process.

As mentioned previously, PR is time consuming, labor intensive, and prone to inter-observer variation. These issues are not a problem with the InterVA model, which provides 100% consistency, minimal effort, and a very short turnaround time in its application to VA interpretation. Additionally, physicians often provide a single cause of death per case, with no place for secondary or underlying causes. In clinical practice, the certification of deaths requires the designation of primary or immediate and secondary or underlying causes of death. The InterVA model is able to provide as many as three possible causes of death, giving it an advantage over the traditional PR process. However, with proper training, physicians who code VA data can easily assess underlying, immediate, and contributing causes of death.

## Conclusions

This study has been able to apply the InterVA model to a large number of VA deaths, unlike previous studies that have used much smaller numbers of deaths. Overall, the performance of the model in VA interpretation was only satisfactory for a few conditions, but they are conditions of great public health significance. Although the model may be limited in its ability to identify COD at the individual level, it has some potential as an innovative community-level tool for identifying COD patterns. This is particularly highlighted in our findings of somewhat comparable CSMFs for the most common COD as interpreted by the model and PR. From a public health perspective, this is useful because the model paints a fairly accurate picture of the disease burden in the study population. Potentially, therefore, this information could be valuable in guiding health policy, programs, and interventions in a resource-constrained and data-deprived setting such as the slums of Nairobi.

However, there is a lot of scope for improving the model and specifically, making allowances for local context-specific features to be built in. A more robust model may be one that, for instance, is tailored to fit a standardized VA questionnaire such as the Sample Vital Registration using Verbal Autopsy (SAVVY) questionnaires developed by the WHO. There is also the small but important issue of how to adequately incorporate multiple probable causes of death generated by the model, which our study did not address. Hence, the next steps will be to refine the model, continue its validation with more extensive data from different settings, and give further thought to the interpretation and analysis of multiple causes of death for individual cases. It will also be useful for the developers of the model to consider programming the model in such a way that it computes uncertainty estimates in the model's predictions. In its current version, the model only presents point predictions, which may be misleading to its users.

## Competing interests

The authors declare that they have no competing interests.

## Authors' contributions

SOO did the literature review and data analysis and wrote the first draft of the manuscript.

CK contributed to the data analysis plan and participated in coding verbal autopsies, writing of the paper and interpretation of the findings.

Both authors read and approved the final manuscript.

## Supplementary Material

Additional file 1 **Spreadsheet showing cause of death categories (including abridged ICD-10 code-list) by physicians and InterVA model**. Click here for file

Additional file 2 **Table showing a comparison of cause of death assessment by PR and most likely cause by InterVA model, for 1,823 verbal autopsies from NUHDSS**. Click here for file
