# Supplementary material for: Verbal autopsy interpretation: a comparative analysis of the InterVA model versus physician review in determining causes of death in the Nairobi DSS
Source: Popul Health Metr. 2010 Jun 29;8:21. doi: 10.1186/1478-7954-8-21 (PMC2902422; doi:10.1186/1478-7954-8-21)
Supplement: Additional file 2 — Table showing a comparison of cause of death assessment by PR and most likely cause by InterVA model, for 1,823 verbal autopsies from NUHDSS. [file 1478-7954-8-21-S2.DOC]

| **Table 1. Comparison of cause of death assessment by PR and most likely cause by InterVA model, for 1823 verbal autopsies  from NUHDSS** | | | | | | | | | | | | | | | | |
| --- | --- | --- | --- | --- | --- | --- | --- | --- | --- | --- | --- | --- | --- | --- | --- | --- |
|  | **InterVA-3 most likely cause►** | | | | | | | | | | | | | | | |
| **Physicians' cause▼** | **Other acute/infectious** | **Malaria** | **Meningitis** | **Pneumonia/Sepsis** | **HIV/AIDS related deaths** | **Pulmonary Tuberculosis** | **Diarrhoeal Disease** | **Maternity related deaths** | **other chronic/NCDs** | **Cardiovascular** | **Injuries/accidents** | **Preterm/perinatal** | **Malnutrition** | **Measles** | **Indeterminate** | **Total** |
|  |  |  |  |  |  |  |  |  |  |  |  |  |  |  |  |  |
| **Other acute/infectious** | **2** | 10 | 2 | 7 | 8 | 0 | 7 | 1 | 4 | 0 | 5 | 1 | 0 | 1 | 14 | 62 |
| **Malaria** | 1 | **15** | 1 | 6 | 4 | 3 | 1 | 0 | 0 | 0 | 0 | 0 | 0 | 1 | 1 | 33 |
| **Meningitis** | 0 | 6 | **20** | 8 | 3 | 1 | 2 | 2 | 2 | 1 | 1 | 0 | 0 | 0 | 11 | 57 |
| **Pneumonia/Sepsis** | 2 | 13 | 8 | **87** | 29 | 14 | 8 | 0 | 2 | 1 | 0 | 2 | 0 | 6 | 27 | 199 |
| **HIV/AIDS related deaths** | 0 | 2 | 2 | 3 | **131** | 203 | 0 | 1 | 3 | 8 | 0 | 0 | 0 | 0 | 66 | 419 |
| **Pulmonary Tuberculosis** | 0 | 0 | 1 | 0 | 9 | **98** | 1 | 0 | 0 | 3 | 0 | 0 | 0 | 0 | 19 | 131 |
| **Diarrhoeal Disease** | 7 | 5 | 4 | 15 | 28 | 1 | **27** | 0 | 0 | 0 | 2 | 0 | 0 | 4 | 24 | 117 |
| **Maternity related deaths** | 0 | 0 | 0 | 0 | 1 | 0 | 0 | **13** | 2 | 1 | 0 | 0 | 0 | 0 | 11 | 28 |
| **other chronic/NCDs** | 2 | 4 | 4 | 10 | 22 | 35 | 1 | 1 | **36** | 20 | 5 | 0 | 0 | 1 | 74 | 215 |
| **Cardiovascular** | 0 | 0 | 1 | 2 | 2 | 5 | 0 | 2 | 2 | **10** | 0 | 0 | 0 | 0 | 9 | 33 |
| **Injuries/accidents** | 0 | 0 | 0 | 6 | 6 | 6 | 0 | 5 | 8 | 2 | **143** | 0 | 0 | 0 | 81 | 257 |
| **Preterm/perinatal** | 0 | 0 | 1 | 22 | 2 | 1 | 0 | 0 | 0 | 0 | 1 | **20** | 0 | 0 | 26 | 73 |
| **Malnutrition** | 0 | 4 | 1 | 4 | 18 | 5 | 0 | 0 | 1 | 0 | 0 | 0 | **0** | 1 | 4 | 38 |
| **Measles** | 0 | 0 | 0 | 7 | 4 | 2 | 0 | 0 | 0 | 0 | 0 | 0 | 0 | **20** | 0 | 33 |
| **Indeterminate** | 3 | 1 | 4 | 10 | 43 | 26 | 1 | 2 | 12 | 9 | 4 | 1 | 0 | 4 | **8** | 128 |
| **Total** | 17 | 60 | 49 | 187 | 310 | 400 | 48 | 27 | 72 | 55 | 161 | 24 | 0 | 38 | 375 | 1823 |
| *Note: The 630/1823 directly corresponding cases are shown in bold text* | | | | | | | | | | | | | | | | |
